# A novel post-percutaneous nephrolithotomy sepsis prediction model using machine learning

**DOI:** 10.1186/s12894-024-01414-x

**Published:** 2024-02-02

**Authors:** Rong Shen, Shaoxiong Ming, Wei Qian, Shuwei Zhang, Yonghan Peng, Xiaofeng Gao

**Affiliations:** 1 Department of Urology, Shanghai Changhai Hospital, No.168 Changhai Rd, Shanghai, 200433 China; 2 Shanghai Institute of Nutrition and Health, Chinese Academy of Sciences, Shanghai, China

**Keywords:** Urinary calculi, Percutaneous nephrolithotomy, Sepsis, Machine learning, Early intervention

## Abstract

**Objectives:**

To establish a predictive model for sepsis after percutaneous nephrolithotomy (PCNL) using machine learning to identify high-risk patients and enable early diagnosis and intervention by urologists.

**Methods:**

A retrospective study including 694 patients who underwent PCNL was performed. A predictive model for sepsis using machine learning was constructed based on 22 preoperative and intraoperative parameters.

**Results:**

Sepsis occurred in 45 of 694 patients, including 16 males (35.6%) and 29 females (64.4%). Data were randomly segregated into an 80% training set and a 20% validation set via 100-fold Monte Carlo cross-validation. The variables included in this study were highly independent. The model achieved good predictive power for postoperative sepsis (AUC = 0.89, 87.8% sensitivity, 86.9% specificity, and 87.4% accuracy). The top 10 variables that contributed to the model prediction were preoperative midstream urine bacterial culture, sex, days of preoperative antibiotic use, urinary nitrite, preoperative blood white blood cell (WBC), renal pyogenesis, staghorn stones, history of ipsilateral urologic surgery, cumulative stone diameters, and renal anatomic malformation.

**Conclusion:**

Our predictive model is suitable for sepsis estimation after PCNL and could effectively reduce the incidence of sepsis through early intervention.

**Supplementary Information:**

The online version contains supplementary material available at 10.1186/s12894-024-01414-x.

## Introduction

Urolithiasis is the most common urinary system disease with a high incidence worldwide [1]. According to surveys, the incidences in North America, Europe, and Asia range from 7 to 13%, 5–9% and 1–5%, respectively [2]. In recent decades, the incidence has been on the rise, causing not only suffering for patients, but also a significant burden on health systems [3].

For complex calculi, such as staghorn calculi, PCNL is the most suitable treatment because of its advantages of high stone removal rate, less surgical trauma, and faster postoperative recovery [4]. However, PCNL is associated with many complications including sepsis, which can affect patient prognosis. Septic shock, which is a serious manifestation of sepsis, significantly increases patient mortality [5].

On the other hand, medical research has entered a new era with the advent of artificial intelligence (AI) [6]. Machine learning is an important branch of AI which is widely used in image recognition and prognosis prediction. For urinary calculi, machine learning is mainly used to assist clinicians in selecting appropriate surgical methods, predicting the success rate of surgery, and determining the composition of the calculi [6–9]. However, no relevant studies have been conducted on the application of machine learning to predict sepsis after PCNL. Therefore, this study aimed to establish a predictive model for sepsis after PCNL using machine learning. This can provide a reference for urologists to identify sepsis and start earlier intervention for high-risk patients.

## Methods

The perioperative data of 694 patients who underwent PCNL treatment at Changhai Hospital between January 2015 and February 2019 were collected, including 404 (58.2%) males and 290 (41.8%) females. All patients provided written informed consent. Urine bacterial cultures were performed on all patients before PCNL. To ensure negative preoperative urine culture results, all positive patients were administered appropriate antibiotic treatment based on culture results. The F22 standard access was used for all PCNL surgery in Changhai Hospital. To avoid data bias caused by different operators, only the surgeries of Professor Gao who is very experienced in PCNL were selected in this study. In case of pyonephrosis, we usually stop the operation immediately after placing the nephrostomy tube. However, for the patients with sufficient antibiotic course and small stone load, or without removing the stone in the main pelvis, simply placing the nephrostomy tube cannot guarantee the drainage effect, we will use ultrasonic negative pressure aspiration, strictly control the operation time, and finish the operation as soon as possible after the removal of stones in the pelvis, and the patient was sent to ICU for intensive care immediately after surgery.

The endpoint of this study was the occurrence of sepsis within 24 h after the operation. Patients were considered to have sepsis when Sequential Organ Failure Assessment (SOFA) score ≥ 2. Due to the early occurrence of sepsis in some patients after surgery, laboratory results could not be obtained in time. Therefore, 22 preoperative and intraoperative variables were used to construct a sepsis predictive model in this study, so that clinicians could judge whether patients would develop sepsis immediately after surgery. Ten continuous variables were used: age, body mass index (BMI), preoperative blood WBC count, creatinine, procalcitonin, bilirubin levels, urinary WBC count, days of preoperative antibiotic use, cumulative stone diameters, and operation time. Twelve classification variables were used: sex, renal anatomical malformation, urinary nitrite, hypertension, diabetes mellitus, isolated kidney, history of ipsilateral urologic surgery, preoperative drainage, preoperative midstream urine bacterial culture, staghorn stones, surgical access, and renal pyogenesis.

To prevent distortion of results with the use of conventional algorithms, we applied the synthetic minority oversampling technique (SMOTE) algorithm to adjust for imbalanced classifications. This algorithm simulated the samples of patients with sepsis and added artificially simulated new samples to the dataset, thus eliminating imbalance in the original data.

Covariance matrix analysis was used to analyse 22 variables, with a redundancy threshold of 0.85. The final model used in this study is a three-layer machine learning framework with mixed super learners. In Layer 1, various machine learning algorithms including Bayesian Classifier, Random Forest, Multi-Gaussian Weighted Classifier and Support Vector Machine were established to minimize the effect of algorithm bias. In Layer 2, meta training was applied by using the prediction results of each trained model in Layer 1 as input features and we obtained mixed super learners to increase predictive performance. The final decision of Layer 3 was the combination of the prediction results in Layer 2 by weighted majority voting. The Monte Carlo cross-validation scheme was applied with 80% training and 20% validation ratios across 100 folds. Each fold had unique training-validation configurations. The Monte Carlo split resulted in 556 samples per fold for the training set. The validation set for each fold contained 69 sepsis samples (139 samples overall). The validation samples were subsampled equally to ensure that none of the label outcomes were over or underrepresented during cross-validation. The predictive performance of the model was evaluated using the Monte Carlo cross-validation scheme with confusion matrix analysis. True-positive, true-negative, false-positive, and false-negative results were calculated by evaluating the validation samples using the established model pipeline in each fold. The sensitivity, specificity, accuracy, positive predictive value, negative predictive value, and area under the curve (AUC) were calculated across each Monte Carlo fold validation results. The selected features and their ranks were calculated across the Monte Carlo folds by Smart Redundancy Reduction, as well as their respective value distributions. The ranks represented the relative importance of the selected features in building the model. The data processing, data analyses, machine learning works and model evaluation were conducted via Python packages including scikit-learn v1.3.2 and imbalanced-learn v0.11.0.

## Results

**Fig. 1:**
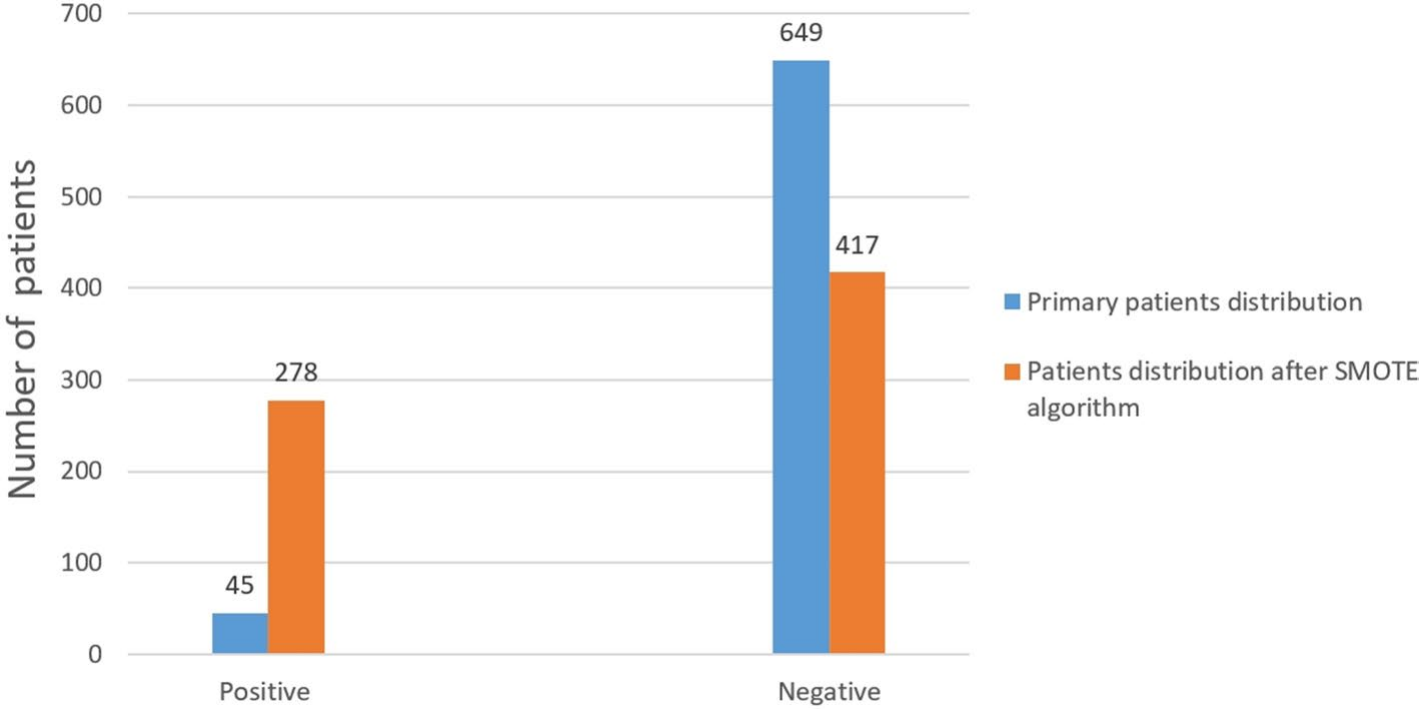
Patients distribution before and after SMOTE algorithm

Baseline characteristics of the all the patients was shown in Table 1. In our study, postoperative sepsis occurred in 45 of 694 patients, including 16 males (35.6%) and 29 females (64.4%). The proportion of patients with and without sepsis was unbalanced (6.5% vs. 93.5%), and after data pre-processing using the SMOTE algorithm, the total number of patients was 695, of which 278 were positive and 417 were negative (40.0% vs. 60.0%). This reduced the interference caused by the low proportion of positive cases in data processing. A comparison of the patient distributions before and after applying the SMOTE algorithm is shown in Fig. 1.

**Table 1:** Baseline characteristics of the all the patients with and without sepsis

	Non-sepsis (*n* = 649)	Sepsis (*n* = 45)	*p*-value
Continuous variables			
Age (years), mean ± SD	52.4 ± 12.6	50.8 ± 12.9	0.411
BMI (kg/m2), mean ± SD	24.2 ± 3.5	23.5 ± 3.4	0.194
Blood WBC (× 10^9^/L), mean ± SD	6.4 ± 1.8	6.0 ± 1.4	0.145
Creatinine (μmoI/L), mean ± SD	89.6 ± 43.8	107.5 ± 61.5	0.010
Procalcitonin (μg/L), mean ± SD	0.05 ± 0.08	0.04 ± 0.03	0.405
Bilirubin (μmoI/L), mean ± SD	10.9 ± 4.7	10.7 ± 4.3	0.781
Urinary WBC (/HP), mean ± SD	117.9 ± 436.6	260.8 ± 935.7	0.056
Days of preoperative antibiotic use (days), mean ± SD	3.5 ± 2.3	4.6 ± 2.6	0.002
Cumulative stone diameters (mm), mean ± SD	55.6 ± 33.0	67.8 ± 34.1	0.017
Operation time (min), mean ± SD	105.0 ± 44.4	107.4 ± 39.6	0.724
Classification variables			
Sex			0.001
Male	388 (59.8%)	16 (35.6%)	
Female	261 (40.2%)	29 (64.4%)	
Renal anatomical malformation			0.071
Positive	44 (6.8%)	0 (0%)	
Negative	605 (93.2%)	45 (100.0%)	
Urinary nitrite			< 0.001
Positive	65 (10.0%)	15 (33.3%)	
Negative	584 (90.0%)	30 (66.7%)	
Hypertension			0.947
Positive	176 (27.1%)	12 (26.7%)	
Negative	473 (72.9%)	33 (73.3%)	
Diabetes mellitus			0.525
Positive	67 (10.3%)	6 (13.3%)	
Negative	582 (89.7%)	39 (86.7%)	
Isolated kidney			0.043
Positive	13 (2.0%)	3 (6.7%)	
Negative	636 (98.0%)	42 (93.3%)	
History of ipsilateral urologic surgery			0.003
Positive	286 (44.1%)	30 (66.7%)	
Negative	363 (55.9%)	15 (33.3%)	
Preoperative drainage			0.064
Positive	104 (16.0%)	12 (26.7%)	
Negative	545 (84.0%)	33 (73.3%)	
Midstream urine bacterial culture			< 0.001
Positive	142 (21.9%)	27 (60.0%)	
Negative	507 (78.1%)	18 (40.0%)	
Staghorn stones			0.011
Positive	106 (16.3%)	14 (31.1%)	
Negative	543 (83.7%)	31 (68.9%)	
Surgical access			0.077
1	554 (85.4%)	34 (75.6%)	
≥2	95 (14.6%)	11 (24.4%)	
Renal pyogenesis			< 0.001
Positive	133 (20.5%)	20 (44.4%)	
Negative	516 (79.5%)	25 (55.6%)

**Fig. 2:**
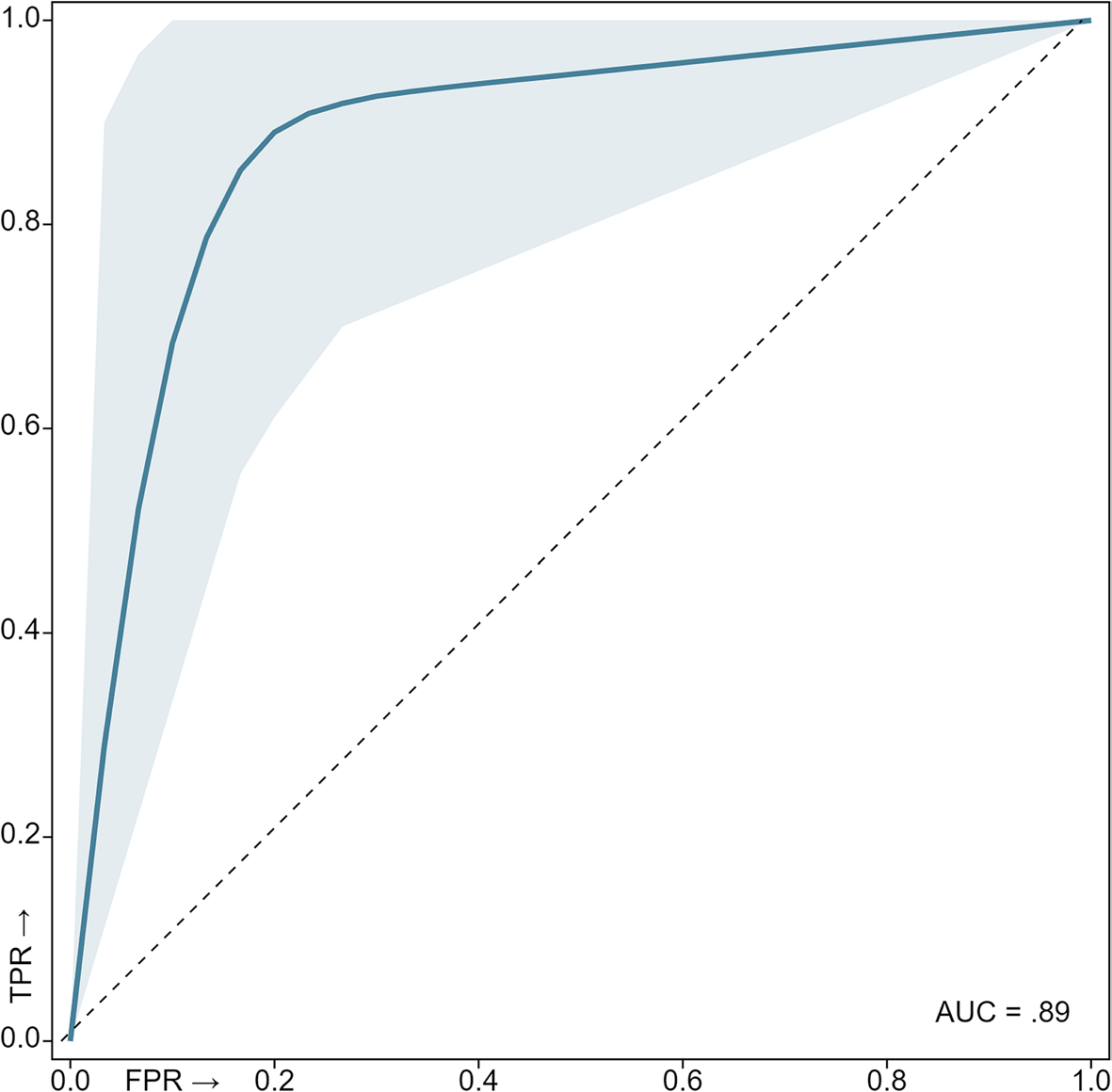
Mean cross-validation ROC curve of the built model. The thick blue line corresponds to the mean ROC curve, while the light blue shaded area represents the spread of all 100 ROC curves generated across the validation folds. FPR – False Positive Rate, TPR – True Positive Rate. Dashed diagonal line represents a reference random-guess AUC for comparison

The sepsis predictive model yielded 87.8% sensitivity, 86.9% specificity, 87.4% accuracy, and 0.89 AUC. Table 2 summarise the Monte Carlo cross-validation performance of all the ensemble prediction models. Figure 2 shows the receiver operating characteristic curve of the predictive model.

**Table 2:** Monte Carlo cross-validation performance of the established model scheme throughout the top-layer prediction model

	Min	LQ	Median	UQ	Max	Mean	Dev
**SNS**	56.66	83.33	90	93.33	100	87.8	6.04
**SPC**	70	83.33	86.66	90	96.66	86.9	4.66
**PPV**	75.67	84.61	87.09	90.47	96.55	87.3	4.11
**NPV**	65.78	83.87	89.28	92.59	100	88.21	5.12
**ACC**	70	85	88.33	90	96.66	87.35	3.58
**AUC**	73.5	85.91	88.72	91.83	97.83	88.6	3.44

*SNS* Sensitivity, *SPC* Specificity, *PPV* Positive Predictive Value, *NPV* Negative Predictive Value, *AC*C Accuracy, *AUC* Area Under the Receiver Operator Characteristics Curve. Performance values are reported as percentages. *LQ* Lower quartile, *UQ* Upper Quartile, *Dev* Deviation

**Fig. 3:**
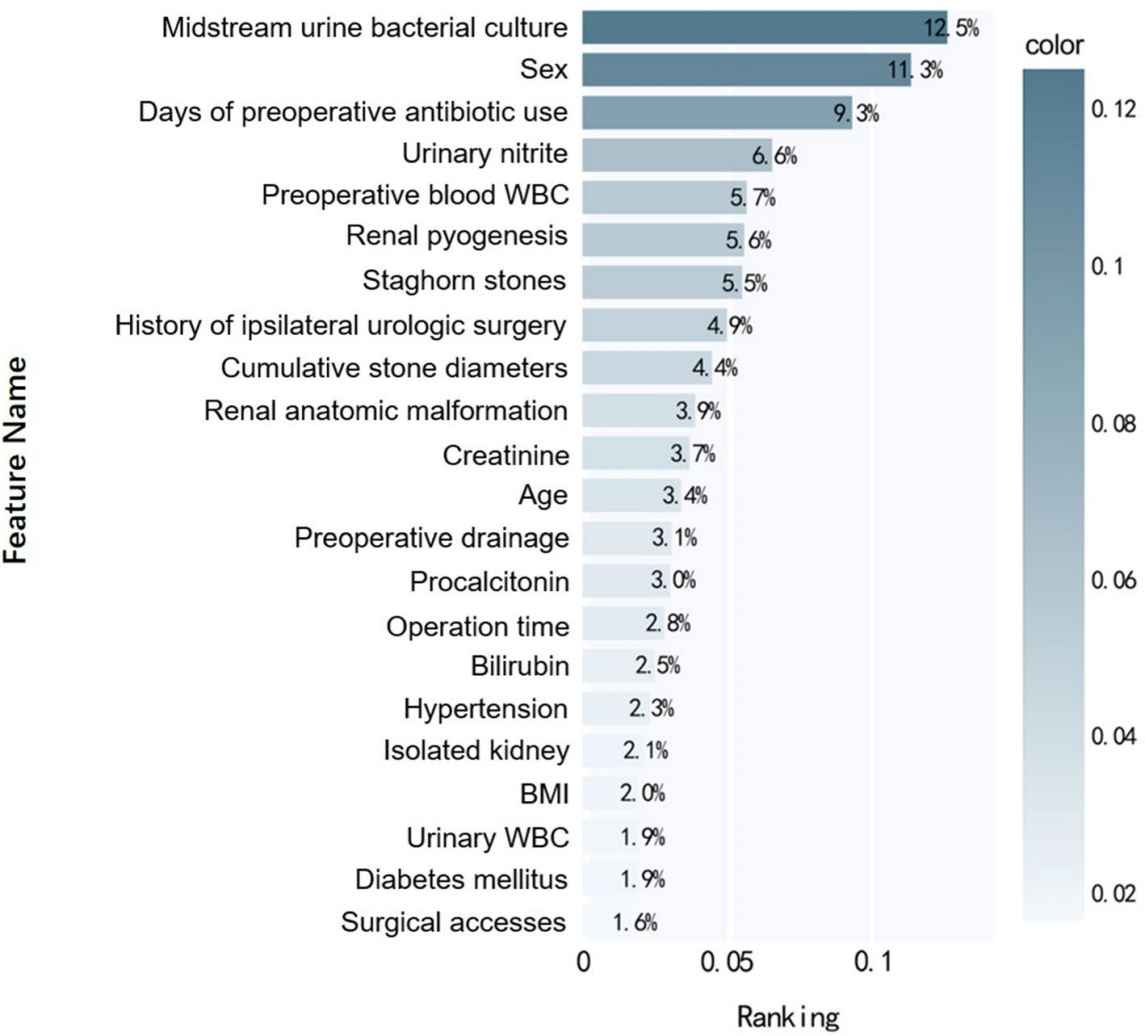
Feature Importance Ranking. Rank values are in percentages

In our sepsis prediction model, the top ten variables were preoperative midstream urine bacterial culture, sex, days of preoperative antibiotic use, urinary nitrite level, preoperative WBC, renal pyogenesis, staghorn stones, history of ipsilateral urologic surgery, cumulative stone diameter, and renal anatomic malformation. Nine of the 10 most relevant features for sepsis prediction originated from the preoperative data. See Fig. 3 for the specific ranking.

## Discussion

In this study, we collected important preoperative and intraoperative clinical data from patients, combined them with machine learning methods, and developed a model that could predict the occurrence of sepsis early after PCNL surgery. The results showed that this model had good predictive efficiency for postoperative sepsis (AUC = 0.89). This can effectively improve the diagnostic ability of urologists for postoperative sepsis in PCNL and reduce the incidence of postoperative adverse events.

Among the common complications of PCNL, infection-based sepsis not only makes treatment more challenging, but also reduces the overall treatment effect [5]. In addition, patients with sepsis have long-term physical, psychological, and cognitive disorders that have a significant negative impact on their long-term prognosis [10]. Furthermore, septic shock, a subset of sepsis, can significantly increase postoperative mortality by affecting the cardiovascular system and cell metabolism [11]. Prevention and early treatment are key to positive outcomes in sepsis; therefore, many studies have focused on exploring the risk factors for sepsis, in the hope of early identification of high-risk patients. According to previous studies, the risk factors for sepsis include age, diabetes, urinary tract infection, stone burden, and positive bacterial cultures of renal pelvic urine and stone [12–15]. However, differences in the study populations, treatment processes, surgical technology, and many other factors in each study led to large discrepancies in the results, making early identification and treatment challenging for clinicians.

Currently, multivariate analysis is the main research method used to assess sepsis risk factors. Logistic regression, a commonly used analysis method, requires normally and linear distributed data with fewer missing points. However, in renal calculi studies, clinical data are easily lost. More importantly, the correlation between several factors limits the application of logistic regression. Compared with these methods, the machine learning does not require linear data and can automatically identify the relationship between variables, allowing analysis even with missing data, which is closer to clinical research. In addition, the algorithm was repeatedly optimised to improve the final predictive ability of the model, rather than simply performing mechanical repetition when processing a large amount of data. There have been some practical applications of artificial intelligence in predicting sepsis after stone surgery. Hong et al. constructed a preliminary screening model for urosepsis based on ultrasound and urinalysis using artificial neural network [16]. This model can provide risk assessment for urosepsis in patients with upper urinary tract calculi, carry out targeted examination or intervention measures, and effectively improve the efficiency of diagnosis and treatment. Considering the low percentage of patients with sepsis, we used the SMOTE algorithm to optimise the data and solve the sample imbalance problem. Furthermore, the parameters included in this study did not exhibit variable repetition owing to a high degree of correlation.

Another advantage of our predictive model is its ability to rank the importance of the variables after data processing. Among the 22 variables included in this study, the top 10 variables contributing to model prediction were preoperative midstream urine bacterial culture, sex, days of preoperative antibiotic use, urinary nitrite, preoperative blood WBC, renal pyogenesis, staghorn stones, history of ipsilateral urologic surgery, cumulative stone diameters, and renal anatomic malformation. Most of the variables with higher importance were consistent with the results of previous studies on risk factors for sepsis. In a prospective single-centre study of 802 patients, Chen et al. found that positive urine culture and the simultaneous positive appearance of urine leukocytes and nitrite were independent risk factors for sepsis [12]. Patel et al. also reported that positive multidrug-resistant urine culture could significantly increase the risk of postoperative infectious complications despite appropriate preoperative antibiotics [17]. Sex is also an important cause of postoperative infections. Previous research showed the incidence of sepsis after PCNL was 4 times higher in female patients than in male patients [12]. In terms of stone burden, Rivera et al. demonstrated that staghorn stones were independently associated with an increased risk of sepsis and that staghorn stones could increase the risk of postoperative infection by more than three times compared to multiple stones [18]. Patel et al. showed that 25% of the patients with postoperative infection events (including sepsis) had renal anatomical abnormalities [17]. In contrast, some studies have shown no correlation between renal anatomical malformations and postoperative infection [19]. Moreover, previous studies also proved that patients with history of ipsilateral surgery are more likely to develop infection events after PCNL [15, 20]. This consistency indicates that machine learning is a process of continuous optimisation and improvement when adjusting parameters. Furthermore, since nine of the 10 most relevant features for predicting sepsis derived from the preoperative data, urologists need to pay more attention to the preoperative clinical data and evaluate patients more comprehensively while adjusting the surgical strategy or intervene as soon as possible after surgery.

This study had some limitations. First, this was a single-centre retrospective study, and the total number of patients with sepsis was relatively small. Even if the SMOTE algorithm was used, the prediction ability of the model would be affected to some extent. Second, different centers may use different references, the variables included in this model were also partly subjective, which may affect the predicting efficiency of the model to some extent. Finally, in the order of importance of variables, the importance of some variables differed from the previous understanding of sepsis risk factors. For example, preoperative blood WBC and creatinine levels were higher than BMI and diabetes. In the following study, we plan to collect cases after 2019 and conduct multi-center studies to increase the number of cases. We will also continue to optimize the inclusion of the variables and strive to further improve the predictive power of the model.

## Conclusions

In conclusion, we established a predictive model for sepsis after PCNL using a machine learning method that provides a reference for urologists in identifying sepsis and could intervene in high-risk patients to effectively reduce the incidence of sepsis.

### Supplementary Information

**Additional file 1: Supplementary Fig. S1. **Correlation matrix view of the data. Correlation value 1 and -1 mean a 100% linear and inverse linear relationship between two features respectively. Feature pairs with near 0 correlation value are considered non-redundant. **Supplementary Table S1. **Preprocessing step algorithms as well as their parameter values performed in all Monte Carlo folds before machine learning. **Supplementary Table S2. **Machine learning (ML) algorithms in the first ML layer with their parameters and value ranges across Monte Carlo (MC) folds. **Supplementary Table S3. **Machine learning (ML) algorithms in the second ML layer with their parameters and value ranges across Monte Carlo (MC) folds. Occurrence of each ML type is represented in percentages across MC folds. **Supplementary Table S4. **Average Monte Carlo (MC) cross-validation performance (%) of ML Layer 1 (ML-1) predictive models as determined by confusion matrix analytics across all MC folds. **Supplementary Table S5. **Average Monte Carlo (MC) cross-validation performance (%) of ML Layer 2 (ML-2) predictive models as determined by confusion matrix analytics across all MC folds. **Supplementary Fig. S2. **Box-plot Monte Carlo (MC) cross-validation performance of the established model scheme throughout the performance of the top-layer prediction model.

## Data Availability

No datasets were generated or analysed during the current study.

## Abbreviations

PCNL: Percutaneous nephrolithotomy
WBC: White blood cell
AI: Artificial intelligence
BMI: Body mass index
SMOTE: Synthetic minority oversampling technique
AUC: Area under the receiver operating characteristic curve
ROC: Receiver operator characteristic
