# Supplementary material for: A novel post-percutaneous nephrolithotomy sepsis prediction model using machine learning
Source: BMC Urol. 2024 Feb 2;24:27. doi: 10.1186/s12894-024-01414-x (PMC10837989; doi:10.1186/s12894-024-01414-x)
Supplement: Supplementary file 1 — Additional file 1: Supplementary Fig. S1. Correlation matrix view of the data. Correlation value 1 and -1 mean a 100% linear and inverse linear relationship between two features respectively. Feature pairs with near 0 correlation value are considered non-redundant. Supplementary Table S1. Preprocessing step algorithms as well as their parameter values performed in all Monte Carlo folds before machine learning. Supplementary Table S2. Machine learning (ML) algorithms in the first ML layer with their parameters and value ranges across Monte Carlo (MC) folds. Supplementary Table S3. Machine learning (ML) algorithms in the second ML layer with their parameters and value ranges across Monte Carlo (MC) folds. Occurrence of each ML type is represented in percentages across MC folds. Supplementary Table S4. Average Monte Carlo (MC) cross-validation performance (%) of ML Layer 1 (ML-1) predictive models as determined by confusion matrix analytics across all MC folds. Supplementary Table S5. Average Monte Carlo (MC) cross-validation performance (%) of ML Layer 2 (ML-2) predictive models as determined by confusion matrix analytics across all MC folds. Supplementary Fig. S2. Box-plot Monte Carlo (MC) cross-validation performance of the established model scheme throughout the performance of the top-layer prediction model. [file 12894_2024_1414_MOESM1_ESM.docx]

**Supplementary Material**

**Data Preprocessing**


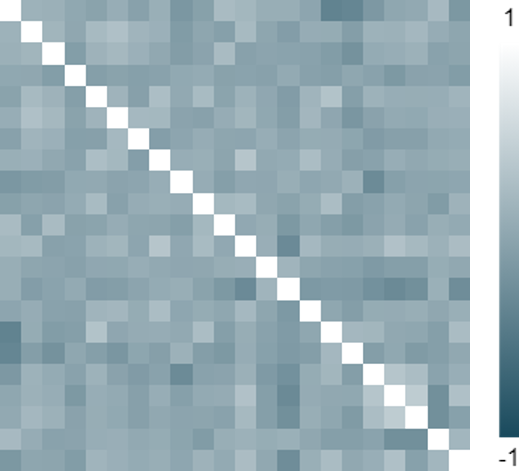


**Supplementary Fig. S1** Correlation matrix view of the data. Correlation value 1 and -1 mean a 100% linear and inverse linear relationship between two features respectively. Feature pairs with near 0 correlation value are considered non-redundant.

**Supplementary Table S1** Preprocessing step algorithms as well as their parameter values performed in all Monte Carlo folds before machine learning. FN - Feature Normalization; KE - Kernel-based Feature Engineering; SRR - Smart Redundancy Reduction.

| Preprocessing step | Algorithm | Parameter | Value |
| --- | --- | --- | --- |
| 1 | FN | Normalization type | Mean-Deviation |
| 2 | KE | Kernels applied | Gaussian; Polynomial; Tanh |
| 3 | FN | Normalization type | Mean-Deviation |
| 4 | SRR | Redundancy Threshold (Covariance) | 0.85 |

**Results**

**Machine Learning Layer 1**

Various machine learning algorithms were established in each fold to minimize the effect of algorithm bias. Each model was trained by randomly selecting 80% of the preprocessed training data per MC fold. For details of the ML algorithms, see Supplementary Table S2.

**Supplementary Table S2** Machine learning (ML) algorithms in the first ML layer with their parameters and value ranges across Monte Carlo (MC) folds. Occurrence of each ML type is represented in percentages across MC folds. BYS – Bayesian Classifier; MGWC – Multi-Gaussian Weighted Classifier; RF – Random Forest Classifier; SVM – Support Vector Machine Classifier;

| ML Algorithm | Parameter | Value Range | Occurrence |
| --- | --- | --- | --- |
| BYS | – | – | 21.94% |
| MGWC | Initial value multiplier | 1 – 10 | 25.68% |
|  | Maximum iterations | 30000 – 150000 |  |
|  | Negative weights allowed | false, true |  |
|  | Scale value multiplier | 0.1 – 50 |  |
|  | Tolerance | 0.00001 – 0.0001 |  |
| RF | Bag fraction | 0.8 – 0.99 | 28.64% |
|  | Bagging method | equalized, normal |  |
|  | Boosting | none, adaboost |  |
|  | Maximum tree depth | 5 – 11 |  |
|  | Minimum samples in leaves | 4 – 8 |  |
|  | Node feature selection method | none |  |
|  | Number of random features per node | 5 |  |
|  | Number of selected trees | 101 – 201 |  |
|  | Number of trees to build | 301 – 1001 |  |
|  | Tree quality metric | gain, gini |  |
|  | Tree selection method | 0 |  |
| SVM | Learning rate | 0.001 – 0.01 | 23.72% |
|  | Maximum iterations | 1000 – 5000 |  |

**Machine Learning Layer 2**

Meta-training sets were created by evaluating the samples of the preprocessed training set in each MC fold by the trained models in ML layer 1. In order to create the meta-training set, the prediction results of each trained model in ML layer 1 were handled as feature values of the given training sample. The meta-training set was the input for training the second ML layer prediction models. These models were trained to identify patterns in the prediction of the first ML layer models to result in mixed super learners. For the parameters of the second layer ML algorithms see Supplementary Table S3.

**Supplementary Table S3** Machine learning (ML) algorithms in the second ML layer with their parameters and value ranges across Monte Carlo (MC) folds. Occurrence of each ML type is represented in percentages across MC folds. MGWC – Multi-Gaussian Weighted Classifier; RF – Random Forest Classifier; SVM – Support Vector Machine Classifier;

| ML Algorithm | Parameter | Value Range | Occurrence |
| --- | --- | --- | --- |
| MGWC | Initial value multiplier | 10 | 33.33% |
|  | Maximum iterations | 5000 – 16000 |  |
|  | Negative weights allowed | true, false |  |
|  | Scale value multiplier | 1 – 5 |  |
|  | Tolerance | 0.0001 |  |
| RF | Bag fraction | 0.8 – 0.99 | 33.33% |
|  | Bagging method | equalized, normal |  |
|  | Boosting | none, adaboost |  |
|  | Maximum tree depth | 5 |  |
|  | Minimum samples in leaves | 4 – 8 |  |
|  | Node feature selection method | none |  |
|  | Number of random features per node | 5 |  |
|  | Number of selected trees | 201 |  |
|  | Number of trees to build | 501 – 1001 |  |
|  | Tree quality metric | gain, gini |  |
|  | Tree selection method | 0 |  |
| SVM | Learning rate | 0.001 – 0.01 | 33.33% |
|  | Maximum iterations | 1000 – 5000 |  |

**Top-Layer Model**

Combination of the prediction results of the second layer ML models was performed by weighted majority voting to provide the final prediction of the model scheme. Weighting of each ML Layer 2 model was calculated based on training performance. In addition, ML Layer 2 models having less training performance than the median of all ML layer 2 model training performances had weight 0 in the final vote.

**Cross-Validation Performance**

Model prediction performance was estimated via the MC cross-validation scheme utilizing confusion matrix analytics. True positive, true negative, false positive and false negative confusion matrix entries were calculated by evaluating the validation samples by the established model pipeline in each fold. Sensitivity, specificity, accuracy, positive predictive as well as negative predictive values were calculated across the MC fold validation results. For the average cross-validation performance of ML Layer 1 and 2 models see Supplementary Table S4 and Supplementary Table S5 respectively. For the cross-validation results of the final (top-layer) prediction models as well as for the summary of the evaluation, see Table 1 and Supplementary Fig. S2.

**Supplementary Table S4** Average Monte Carlo (MC) cross-validation performance (%) of ML Layer 1 (ML-1) predictive models as determined by confusion matrix analytics across all MC folds. BYS – Bayesian Classifier; MGWC – Multi-Gaussian Weighted Classifier; RF – Random Forest Classifier; SVM – Support Vector Machine Classifier; SNS – Sensitivity; SPC – Specificity; PPV – Positive Predictive Value; NPV – Negative Predictive Value; ACC – Accuracy; OCC – Occurrence. Performance and occurrence values are in percentages.

|  | SNS | SPC | PPV | NPV | ACC | OCC |
| --- | --- | --- | --- | --- | --- | --- |
| BYS | 92 | 42 | 63 | 87 | 67 | 22 |
| MGWC | 62 | 79 | 76 | 69 | 71 | 26 |
| RF | 90 | 82 | 84 | 90 | 86 | 29 |
| SVM | 76 | 73 | 74 | 76 | 75 | 24 |

**Supplementary Table S5** Average Monte Carlo (MC) cross-validation performance (%) of ML Layer 2 (ML-2) predictive models as determined by confusion matrix analytics across all MC folds. MGWC – Multi-Gaussian Weighted Classifier; RF – Random Forest Classifier; SVM – Support Vector Machine Classifier; SNS – Sensitivity; SPC – Specificity; PPV – Positive Predictive Value; NPV – Negative Predictive Value; ACC – Accuracy; OCC – Occurrence. Performance and occurrence values are in percentages.

|  | SNS | SPC | PPV | NPV | ACC | OCC |
| --- | --- | --- | --- | --- | --- | --- |
| MGWC | 83 | 78 | 80 | 83 | 81 | 33 |
| RF | 88 | 87 | 87 | 88 | 87 | 33 |
| SVM | 84 | 70 | 74 | 82 | 77 | 33 |


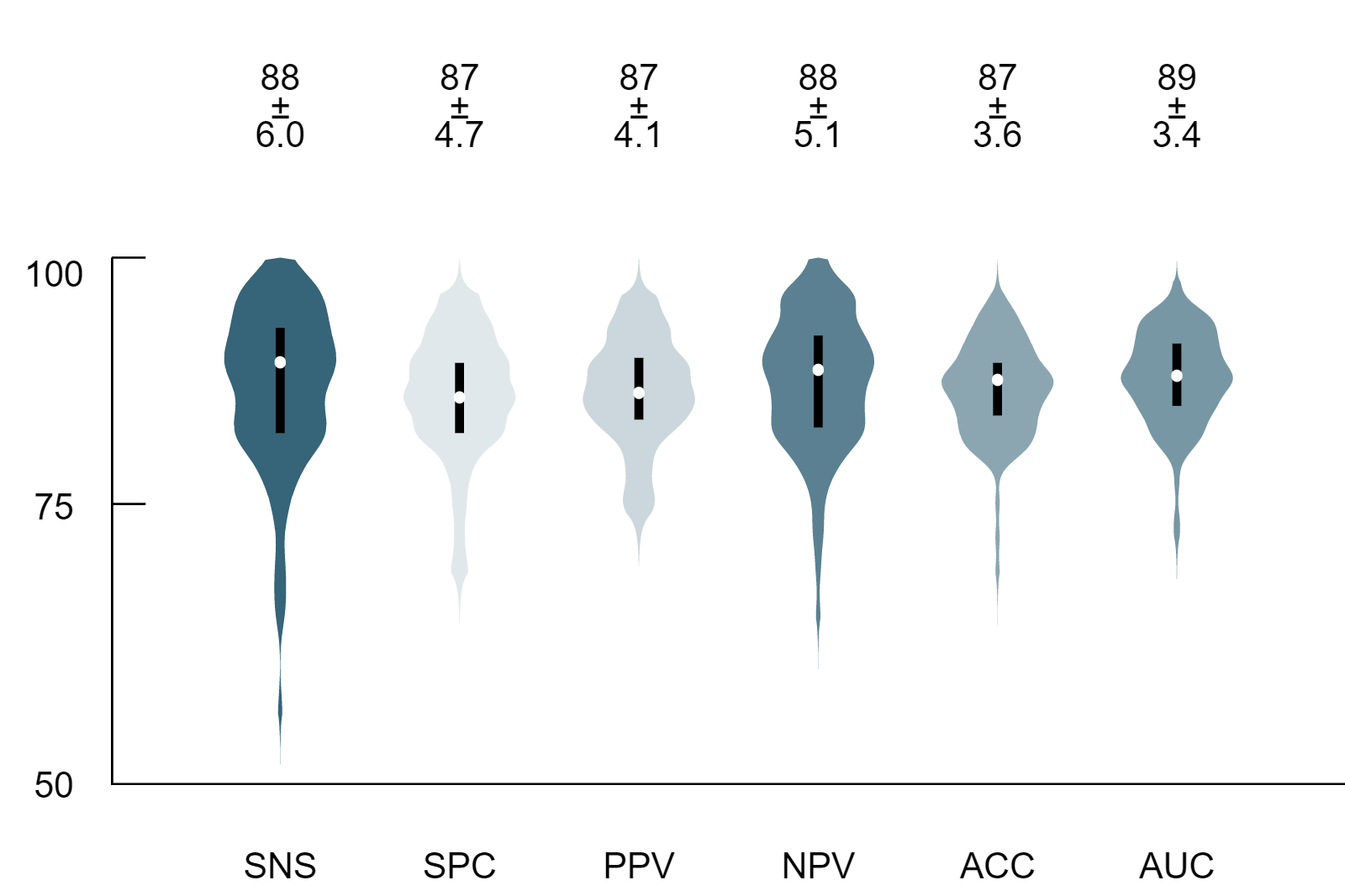


**Supplementary Fig. S2** Box-plot Monte Carlo (MC) cross-validation performance of the established model scheme throughout the performance of the top-layer prediction model. Performance values were determined by confusion matrix analytics across all MC folds. SNS – Sensitivity; SPC – Specificity; PPV – Positive Predictive Value; NPV – Negative Predictive Value; ACC – Accuracy; AUC – Area Under the Receiver Operator Characteristics Curve. Performance values are in percentages.
